# Loss of homeostatic microglial phenotype in *CSF1R-*related Leukoencephalopathy

**DOI:** 10.1186/s40478-020-00947-0

**Published:** 2020-05-19

**Authors:** Liam Kempthorne, Hyejin Yoon, Charlotte Madore, Scott Smith, Zbigniew K. Wszolek, Rosa Rademakers, Jungsu Kim, Oleg Butovsky, Dennis W. Dickson

**Affiliations:** 1 Department of Neuroscience, Mayo Clinic, 4500 San Pablo Road, Jacksonville, FL 32224 USA; 2 Institute of Neurology, University College London, London, UK; 3 Neurobiology of Disease Graduate Program, Mayo Clinic Graduate School of Biomedical Sciences, Jacksonville, FL USA; 4 Center for Neurologic Diseases, Department of Neurology, Brigham and Women’s Hospital, Harvard Medical School, Boston, MA USA; 5 Department of Neurology, Mayo Clinic College of Medicine, Jacksonville, FL USA; 6 Evergrande Center for Immunologic Diseases, Brigham and Women’s Hospital, Harvard Medical School, Boston, MA USA

**Keywords:** *CSF-1R*-related leukoencephalopathy, Hereditary diffuse leukoencephalopathy with spheroids (HDLS), Adult leukoencephalopathy with spheroids and pigmented glia (ALSP), CSF-1R, CSF-1, Microglia, Immunohistochemistry, RNA expression profiling

## Abstract

Microglia are resident macrophages of the central nervous system, and their unique molecular signature is dependent upon CSF-1 signaling. Previous studies have demonstrated the importance of CSF-1R in survival and development of microglia in animal models, but the findings are of uncertain relevance to understanding the influence of CSF-1R on microglia in humans. Hereditary diffuse leukoencephalopathy with spheroids (HDLS) [also known as adult onset leukoencephalopathy with spheroids and pigmented glia (ALSP)] is a neurodegenerative disorder primarily affecting cerebral white matter, most often caused by mutations of *CSF1R*. Therefore, we hypothesized that the molecular profile of microglia may be affected in HDLS. Semi-quantitative immunohistochemistry and quantitative transcriptomic profiling revealed reduced expression of IBA-1 and P2RY12 in both white and gray matter microglia of HDLS. In contrast, there was increased expression of CD68 and CD163 in microglia in affected white matter. In addition, expression of selective and specific microglial markers, including P2RY12, CX3CR1 and CSF-1R, were reduced in affected white matter. These results suggest that microglia in white matter in HDLS lose their homeostatic phenotype. Supported by gene ontology analysis, it is likely that an inflammatory phenotype is a key pathogenic feature of microglia in vulnerable brain regions of HDLS. Our findings suggest a potential mechanism of disease pathogenesis by linking aberrant CSF-1 signaling to altered microglial phenotype. They also support the idea that HDLS may be a primary microgliopathy. We observed increased expression of CSF-2 in gray matter compared to affected white matter, which may contribute to selective vulnerability of white matter in HDLS. Our findings suggest that methods that restore the homeostatic phenotype of microglia might be considered treatment approaches in HDLS.

## Introduction

Macrophages are a diverse family of phagocytic effector cells of innate immunity. They play vital roles in defense against pathogens as well as clearance of tissue debris and repair of damaged tissue [13]. Microglia are a unique population of macrophages of the central nervous system populated in early gestation from yolk sac-derived precursor cells, with replenishment by local proliferation rather than influx of blood borne cells [41, 42]. In addition to their immune functions, microglia play important roles in synaptic development and myelin maintenance [21, 46]. Recent studies have identified a specific molecular and functional signature of microglia, separating them from macrophages as well as other brain cell types [6, 7, 20, 27]. Moreover, this unique molecular signature is dependent upon CSF-1 [7]. A number of animal studies have demonstrated a role for CSF-1R in survival and development of macrophages [10, 54, 55]. Many of these studies were limited in exploring functional relationships between CSF-1 signaling and microglia due to use of reagents that did not distinguish macrophage subtypes or by incomplete inhibition of CSF-1 signaling. Limited functional studies of CSF-1 signaling associated with human pathology have been reported [50].

It has been discovered that autosomal dominant mutations in *CSF1R,* resulting in partial loss of function in CSF-1R signaling, are the major genetic cause of hereditary diffuse leukoencephalopathy with spheroids (HDLS) [22, 40, 44]. HDLS, also known as adult-onset leukoencephalopathy with spheroids and pigmented glia (ALSP) [23], is a progressive neurodegenerative disorder characterized by cerebral white matter degeneration with axonal spheroids, ballooned cortical neurons, reactive astrocytosis, and lipid- and pigment-laden macrophages [2, 3, 28]. Recessive mutations in alanyl-transfer (t) RNA synthetase 2 (AARS2) can produce a similar disorder, through presumably different mechanisms [29]. There are also families with nearly identical antemortem clinical findings confirmed and HDLS-like pathology at autopsy without mutations in either CSF1R or AARS2 [Wszolek, ZK, unpublished]. CSF-1R is the main receptor for CSF-1 (also known as M-CSF), which is one of two major macrophage trophic factors, with CSF-2 (also known as GM-CSF) being the other [26]. CSF-1R signaling mediates important cues for survival, proliferation, differentiation and activation of cells of the macrophage lineage, including microglia [17].

In light of current evidence, we studied functional properties of microglia in HDLS. In this study, we investigated molecular and pathological effects of loss of CSF-1 signaling due to mutations in *CSF1R* in postmortem brain tissues of 11 patients with HDLS. We assessed macrophage and microglial populations with immunohistochemistry for various cell type markers. We also analyzed gene expression changes, focusing on genes associated with macrophage and microglial functionality. Our findings suggest a potential mechanism of disease pathogenesis that links aberrant CSF-1 signaling to altered microglial phenotype and to the selective vulnerability of white matter in HDLS.

## Materials and methods

### Case materials

All leukoencephalopathy cases used in this study were diagnosed as HDLS and confirmed to have *CSF1R* mutations, and we therefore use the more specific nomenclature of HDLS, rather than ALSP. All HDLS cases were submitted to the neurodegenerative disorders brain bank at Mayo Clinic, Jacksonville. Inclusion criteria for morphologic studies were presence of formalin-fixed, paraffin-embedded tissue of HDLS (*n* = 11) and controls without significant neuropathologic changes (*n* = 11) (Table 1). Assigned case numbers for expression studies were randomly chosen. Many of the HDLS cases have been previously reported or included in genetic studies of HDLS [3, 33, 44].

**Table 1:** Demographic and genetic features of HDLS and control case cohorts. Inclusive of cases with fixed tissue available (+) for imaging studies as well as cases from enlarged HDLS cohort without fixed tissue available (−) with available pathology reports and demographic data sufficient for inclusion in gender and AD pathology analyses. NA = information not available or not applicable to that case. Student’s t-test was performed on age at death between male and female HDLS cohorts, female age at death was significantly younger than male (*p* = 0.032)

Group	Case #	Sex	Age	*CSF1R* Mutation	Histopathology	Expression
HDLS	1	F	46	c.1897G > A, p.Glu633Lys	+	+
HDLS	2	M	55	c.2320-2A > G, p.Cys774_Asn814del	+	+
HDLS	5	F	58	c.2633C > A p.Pro878His	+	+
HDLS	6	M	49	c.2297 T > C, p.Met766Thr	+	+
HDLS	9	F	52	c.2381 T > C, p.Ile794Thr	+	+
HDLS	11	M	51	c.2330G > A, p.Arg777Gln	+	+
HDLS	13	M	71	c.1897G > A, p.Glu633Lys	+	–
HDLS	14	F	49	c.2603 T > C, p.Lys868Pro	+	–
HDLS	15	F	55	c.2603 T > C, p.Lys868Pro	+	–
HDLS	16	M	62	c.2381 T > C, p.Ile794Thr	+	–
HDLS	17	F	NA	c.2297 T > C, p.Met766Thr	+	–
Normal	3	M	69		–	+
Normal	4	M	63		–	+
Normal	7	F	61		–	+
Normal	8	F	60		+	+
Normal	10	M	53		–	+
Normal	12	F	56		–	+
Normal	18	M	74		+	–
Normal	19	M	51		+	–
Normal	20	F	65		+	–
Normal	21	M	82		+	–
Normal	22	F	60		+	–
Normal	23	M	65		+	–
Normal	24	M	50		+	–
Normal	25	M	50		+	–
Normal	26	F	53		+	–
Normal	27	M	63		+	–

### Immunohistochemistry and immunofluorescence

Immunohistochemistry for IBA-1 (019–19,741, 1:3000, Wako Chemicals, VA, USA), CD68 (M0814, 1:1000, DAKO, CA, USA), CD163 (NCL-L-10D6, 1:250, Leica (Novocastra), Newcastle, UK), and P2RY12 (1:250, Dr. Butovsky) was performed on sections of medial frontal lobe that included periventricular white matter, corpus callosum and anterior cingulate gyrus. For comparison, we used horizontal sections of cerebellum at the level of the dentate nucleus. Immunohistochemistry was performed on glass-mounted, 5-μm thick formalin-fixed, paraffin-embedded sections. Sections were deparaffinized in three 5 min washes of xylene, rehydrated in three 2 min washes of a graded series of ethanol (100, 100, 95%), and washed thoroughly in dH_2_0 prior to steaming in either dH_2_0 or pH 6 citrate buffer (depending on the antibody) for antigen retrieval. All stains were processed by a DAKO AutostainerPlus (DAKO, Carpinteria, CA, USA) with the DAKO EnVision™ + System-HRP (diaminobenzidine) secondary antibody system. Normal goat serum (1:20 in Tris buffered saline; Sigma, St. Louis, MO, USA) was used to block nonspecific antibody binding.

Double-labelled immunofluorescence was performed with IBA-1 (1:750) and CD68 (1:500) on sections of periventricular white matter at the level of the anterior cingulate gyrus. Slides were deparaffinized, rehydrated, and washed as described above. They were then blocked with DAKO serum-free protein block prior to incubation with a cocktail of the primary antibodies over night at 4 °C. After briefly washing with phosphate buffered saline, slides were incubated with a cocktail of Alexa Fluor 488 and 568 conjugated secondary antibodies (1:500, Molecular probes, Eugene, OR, USA) for 1.5 h at room temperature. Prior to coverslipping, the slides were treated with Sudan Black for 2 mins to block autofluorescence. Slides were cover slipped with Vectashield with DAPI mounting media (Vector Laboratories, Burlingame, CA, USA). Images were taken using a Zeiss Axio Imager Z1 microscope (Carl Zeiss Microscopy, Jena, Germany).

### Image analysis

Immunostained sections were converted into high-resolution digital images with Aperio ScanScope XT Bright field slide scanner (Aperio Technologies, Vista, CA, USA). Uniform-sized regions (2 × 480,000 μm^2^ regions) of acute white matter lesions, chronic white matter lesions, deep peri-lesional gray matter (layers IV, V & VI), and superficial peri-lesional gray matter (layers I, II & III) were selected for each different immunostain for each case and tissue section. Custom designed algorithms were developed using Aperio ImageScope software to optimize signal-to-noise for quantification of each antibody.

### Gene expression array

A customized gene expression array chip was designed on quantitative NanoString nCounter platform (NanoString Technologies, Seattle, WA, USA). The customized MG447 human microglia chip contains 376 microglial transcripts, 40 inflammation related transcripts, 6 positive reference genes and 8 negative controls in each platform [7]. Normal controls (*n* = 6) and HDLS patients (*n* = 6) were selected based on availability of age- and sex-matched brain samples. The brain samples were collected from frontal white matter, frontal neocortex and cerebellar white matter. Total RNA was extracted from brain samples using Trizol™ reagent (Invitrogen, Carlsbad, CA, USA) according to the manufacturer’s instruction. 100 ng of total RNA from each sample was used for the nCounter analysis according to the manufacturer’s protocol. All analyses were randomized in terms of diagnostic category and brain region and analyses were double-blinded. One subject in the control group (subject #3), had to be excluded from analyses given that it was an outlier based on heat map and principal component analyses (PCA).

### Expression data analysis

Gene expression data were normalized against 6 housekeeping genes and positive controls. The expression data were excluded when they had lower than average of background signal from negative controls. PCA plots and heat maps were generated using Partek® Genomics Suite® software (Partek Inc. St. Louis, MO, USA) with Euclidean and Average Linkage for clustering methods. Differentially expressed transcripts were analyzed using one-way analysis of variance (ANOVA) in Partek® Genomics Suite® software. Enrichment analysis was performed using MetaCore™ (GeneGo, St. Joseph, MI, USA).

### Statistical analyses

SigmaPlot version 11 (Systat Software, San Jose, CA, USA) was used for statistical analyses of immunohistochemistry data. Due to small sample sizes, Kruskal-Wallis ANOVA on rank was performed with Dunn’s post-hoc analysis for pairwise comparison between HDLS, normal controls, and other white matter diseases for each area analyzed. Data that passed Shapiro-Wilk normality test were subjected to one-way ANOVA followed by Holm-Sidak pairwise post-hoc analysis. Mann-Whitney U test was used to compare two groups of data. GraphPad Prism 6.00 for Windows (GraphPad Software, La Jolla, CA, USA) was used for graphical presentation. The following symbols were used to indicate the degree of significant difference: * *P* < 0.05, ** *P* < 0.01, *** *P* < 0.001.

## Results

### Alterations in microglial and macrophage phenotypes in HDLS

**Fig. 1:**
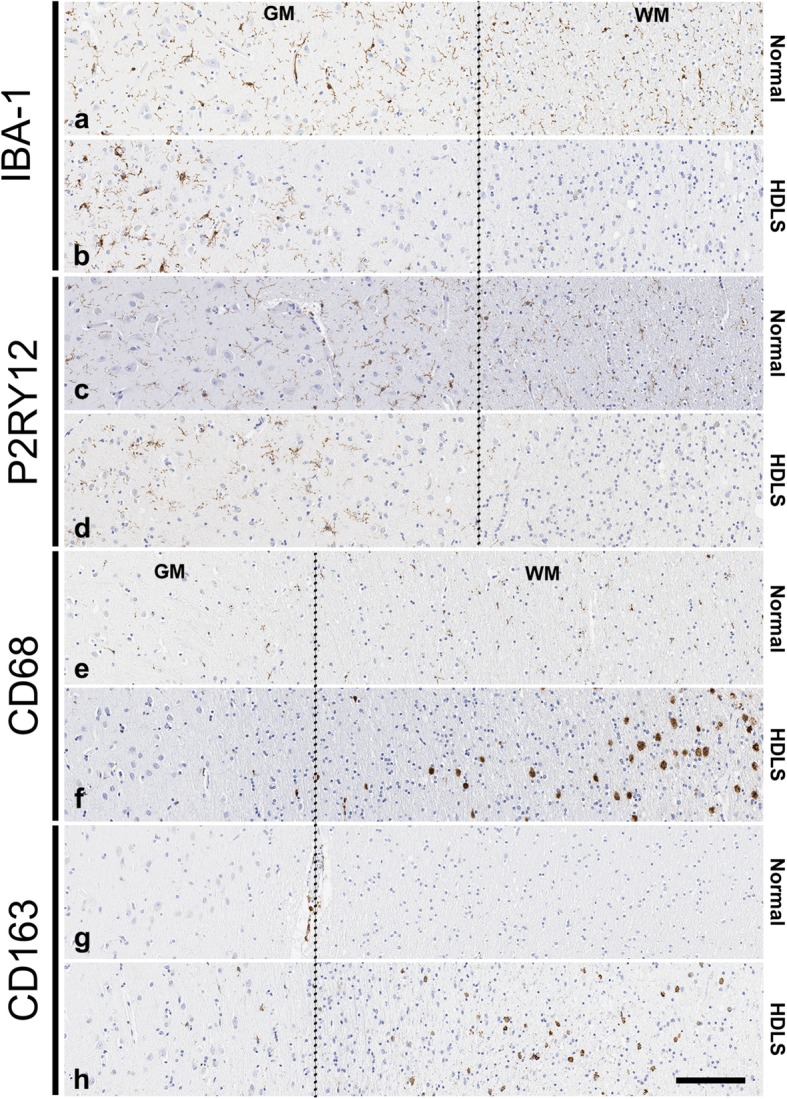
Alterations in microglial and macrophage phenotype in HDLS. Microscopic comparison of 4 macrophage markers in a representative HDLS case (case #7) and a representative normal control (case #8). IBA-1 expression (**a** & **b**) is decreased in the white matter of HDLS. Similarly, P2RY12 expression (**c** & **d**) is decreased in white matter of HDLS. Many amoeboid CD68 (**e** & **f**) macrophages are observed in HDLS lesional white matter. Presence of parenchymal CD163 (**g** & **h**) positive cells are confined to the lesional white matter of HDLS, with almost no cells detected seen in normal parenchyma. GM = gray matter, WM = white matter. Dotted line separates gray and white matter. Scale bar = 100 μm

The focus of this study is on a series of patients with *CSF1R-*related leukoencephalopathy consistent with HDLS [2]. ALSP is an alternative term proposed by some for cases with similar pathology [23], but ALSP includes cases without *CSF1R* mutations. Mutations in *CSF1R* cause HDLS, and CSF-1R-mediated signaling is critical for microglial development. Therefore, to assess changes in microglia in HDLS, we performed immunohistochemistry with a panel of macrophage and microglial markers, including IBA-1, P2RY12, CD163 and CD68. We compared findings in affected cerebral white matter of HDLS at the level of the anterior cingulate gyrus with a similar region of neuropathologically normal controls. We also analyzed adjacent cerebral cortex and unaffected cerebellar white matter of HDLS. We used IBA-1 as a pan-macrophage marker [1]. In affected white matter of HDLS, IBA-1 immunoreactivity was almost undetectable, but relatively spared in adjacent cortex (Fig. 1a and b). More interestingly, the distribution of IBA-1 positive cells in white and gray matter was variable, including areas in deeper white matter with no IBA-1 positive cells and other areas, closer to the gray-white junction, with seemingly increased density of IBA-1 positive cells. Since both macrophages and microglia express IBA-1, we also studied expression of P2RY12, a purinergic receptor increasingly considered to be a specific microglia marker [7]. The staining pattern of P2RY12 was similar to that of IBA-1, with loss of staining in affected white matter and relative sparing in the adjacent neocortical gray matter (Fig. 1c and d). P2RY12 immunoreactivity in neocortical gray matter had a similar distribution pattern as IBA-1 immunoreactivity.

To understand whether the decrease of IBA-1 and P2RY12 immunoreactivity was due to a decrease in the number of microglia or changes in their phenotype, we also studied adjacent sections of the same cases with CD68 immunohistochemistry. CD68 detects a lysosomal antigen in inflammatory or amoeboid macrophages and microglia [4]. Despite the loss of IBA-1 and P2RY12 immunoreactive microglia, there were many CD68-positive macrophages in affected white matter (Fig. 1e and f).

The loss of IBA-1 and P2RY12 immunoreactivity with retention of CD68 immunoreactivity in affected HDLS white matter raised the question of whether the CD68-positive cells were parenchymal microglia or peripheral monocyte-derived macrophages. To address this question, we investigated immunoreactivity of CD163. CD163 is a hemoglobin-haptoglobulin scavenger receptor that is considered to be expressed exclusively on perivascular macrophages in the brain under normal conditions, as well as peripheral cells of monocyte lineage [38, 56]. Interestingly, there were many parenchymal CD163-positive cells in affected white matter of HDLS (Fig. 1g and h). This suggests that at least a subpopulation of macrophages in HDLS white matter may be peripheral monocyte-derived cells. Taken together, alterations in macrophage markers seem to indicate the possibility of an altered phenotype of resident microglia and/or migration of peripheral monocytes into affected white matter of HDLS.

### Analysis of microglial phenotypes in different cortical layers

We observed more marked white matter pathology, including axonal loss, tissue vacuolation and gliosis, in deep white matter compared with the white matter near the gray-white junction (i.e. arcuate fibers). We also noted that white matter near the cortical gray-white junction had many axonal spheroids, frequent lipid-laden macrophages, relatively intact oligodendroglia, and less myelin loss [3]. Thus, we separately analyzed deep white matter and subcortical white matter to investigate the association between disease severity and microglial phenotypes; we also compared affected cerebral white matter to unaffected cerebellar white matter. Given evidence from a *Csf1r*^*+/−*^ mouse model that deep cortical layers may be more severely affected than superficial cortical layers [9], we also analyzed superficial cortical layers (I, II and III) and deep cortical layers (IV, V and VI) separately.

**Fig. 2:**
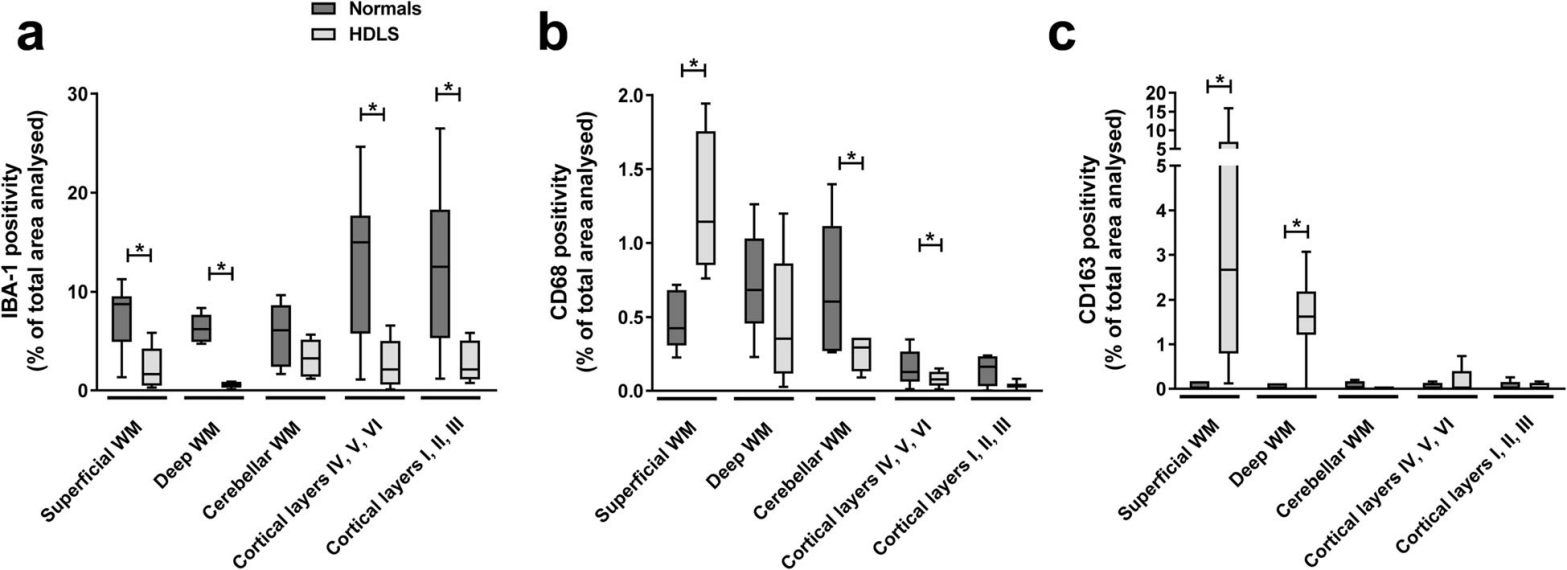
Analysis of microglial phenotypes in different cortical layers. Graph panel of quantified (with Aperio software and specifically designed algorithms) DAB-based immunohistochemistry images of: macrophage markers IBA-1 (**a**), CD68 (**b**), CD163 (**c**) in HDLS (*n* = 12 for all stains and regions) and Normals (*n* = 12 for all stains and regions). Data represented as Tukey Box and Whisker plots. * indicates statistically significant *p*-value< 0.05

Semiquantitative analysis of IBA-1 expression in these brain regions revealed significantly decreased IBA-1 immunoreactivity in both subcortical and deep white matter and in both superficial and deep cortical layers of HDLS compared with normal controls (Fig. 2a). In contrast, CD68 immunoreactivity was most marked in superficial cerebral white matter and less in deep white matter and in cerebellar white matter of HDLS (Fig. 2b). Taken together, the decrease in IBA-1 and CD68 in superficial and deep gray matter is suggestive of a decrease in gray matter microglial cells, as well as the previously described non-uniform distribution.

We also observed significant increase in CD163 immunoreactive cells in both superficial and deep white matter of HDLS (Fig. 2c), but minimal CD163 cells in any region of controls. These semi-quantitative analyses suggest either an altered phenotype of resident microglia or influx of peripheral monocytes into both superficial and deep white matter of HDLS.

### Analysis of subpopulations of macrophages in HDLS

**Fig. 3:**
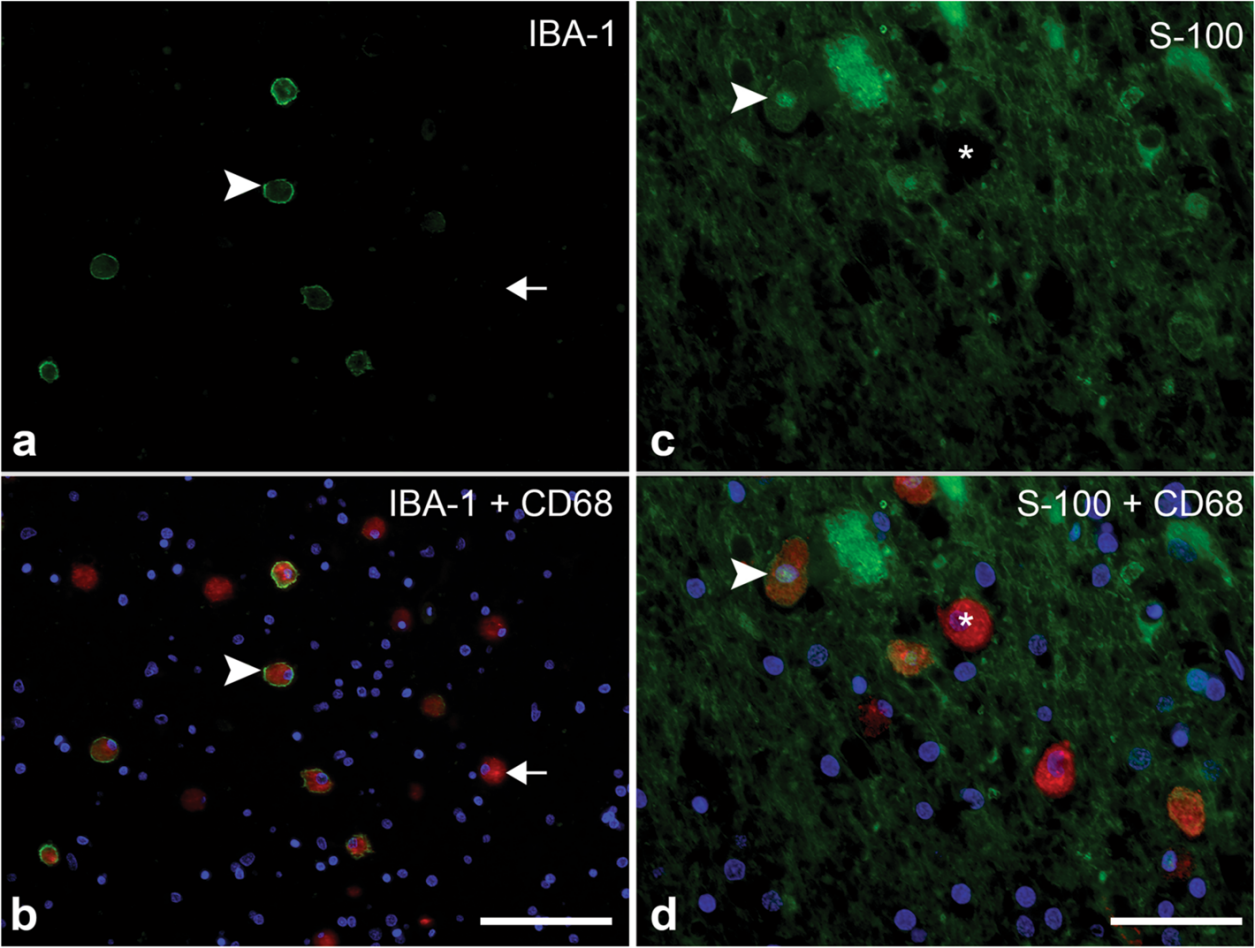
Analysis of sub-population of macrophages in HDLS. **a** & **b** Immunofluorescence double stain of CD68 (Alexa Fluor 568 (red)), and IBA-1 (Alexa Fluor 488 (green)) with DAPI nuclear staining (blue). White arrowhead indicates an example of an IBA-1, CD68 double labelled amoeboid macrophage. White arrow indicates an example of a CD68 positive, IBA-1 negative amoeboid cell. Scale bar = 50 μm. **c** & **d** Immunofluorescent double stain of CD68 (Alexa Fluor 568 (red)), and S-100 (Alexa Fluor 488 (green)), with DAPI nuclear staining (blue). White arrowhead indicates an example of a CD68, S-100 double labelled cell with nuclear and cytoplasmic S-100 expression. Asterisk indicates example of a CD68 positive, S-100 negative cell. Scale bar = 25 μm

Based on these observations of macrophage markers in HDLS, we hypothesized that partial loss of the CSF-1 signaling may cause either abnormal differentiation of microglia or migration of monocytes into affected white matter, or both. Since CSF-2 is known to promote differentiation of myeloid cells into cells of dendritic lineage, possible imbalance between CSF-1 and CSF-2 activity in HDLS may favor differentiation of progenitor cells into dendritic cells [12]. Therefore, double immunofluorescence for IBA-1 with CD68 and for CD68 with S-100 was used to further characterize IBA-1-negative macrophages in superficial white matter. The presence of CD68-positive, IBA-1-negative macrophages in the white matter of HDLS was confirmed (Fig. 3a and b). While S-100 is often used as an astrocytic marker [48, 49], it is also expressed in both mature and immature dendritic cells, such as Langerhans cells of the skin [47, 51]. S-100 and CD68 double immunofluorescence revealed S-100-positive and CD68-positive cells (Fig. 3c and d). These results suggest a sub-population of the infiltrating myeloid-derived cells are aberrantly differentiating into cells of a dendritic lineage that are not normally present in the brain parenchyma.

### Identification of region-specific changes microglial transcriptome in HDLS

**Fig. 4:**
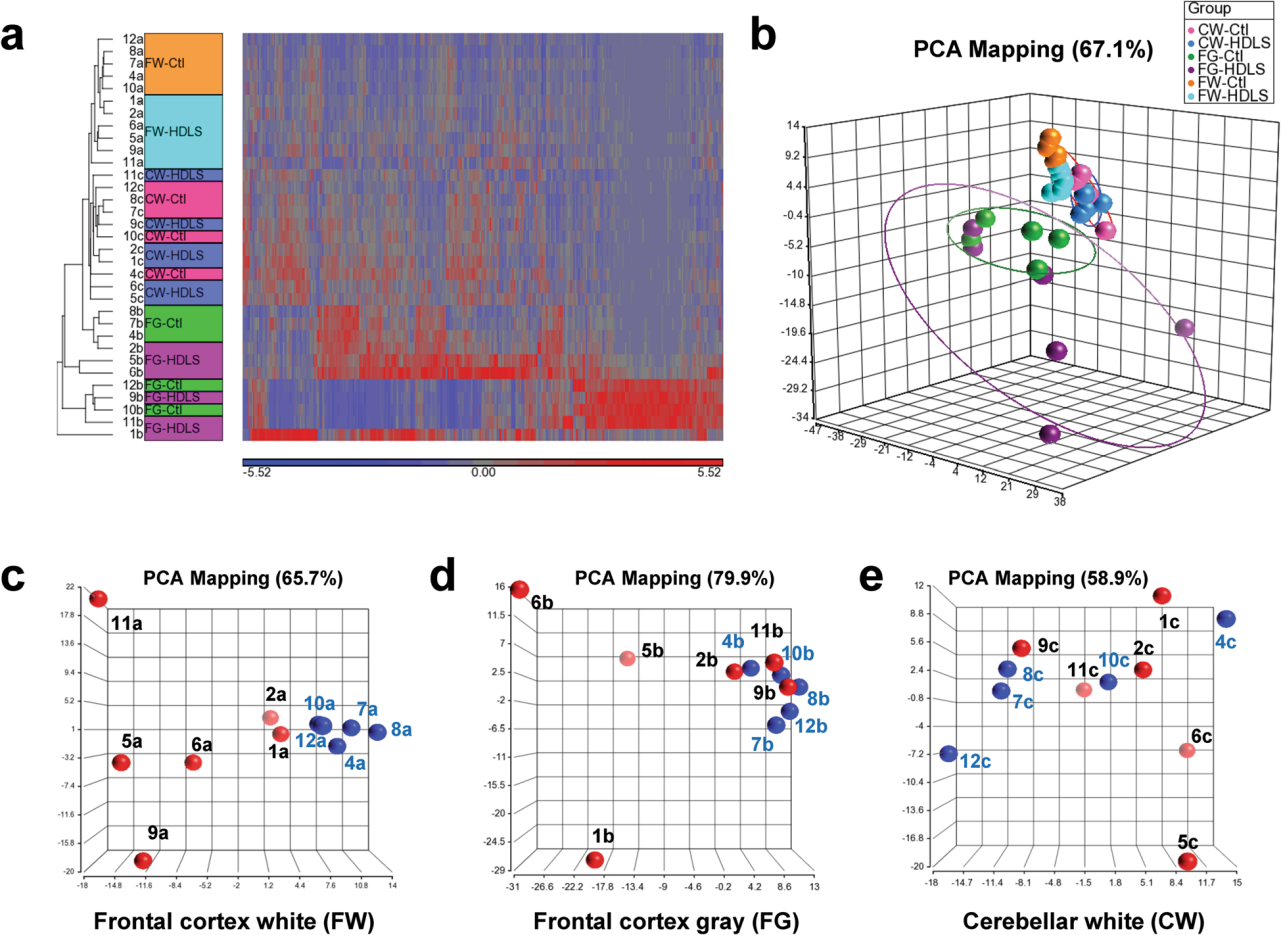
Identification of brain region-specific transcriptional changes in HDLS. (a & b) Heat map and PCA map of total transcription profiles in different brain regions. The same color codes were used to display the same groups in (a) and (b). (a) The heat map shows that HDLS-cases are clearly separated from control-cases by their molecular signatures in frontal cortex white matter. Up- and down-regulated transcripts in HDLS are shown in red and blue, respectively. (b) The PCA components from FW are proximal to the CW than FG indicating the similarity in cellular structure-specific gene expression patterns. PCA 67.1%, PC1 = 33.3%, PC2 = 24%. (c-e) PCA analysis of total transcripts in each brain areas, FW, FG and CW. Control cases (*n* = 5) are shown in blue, and HDLS cases (*n* = 6) are shown in red marbles. FW, FG and CW samples were acquired from the same individual assigned with the number from 1 to 12 by individual, and assigned with the alphabet a-c by brain area. (c) PCA map of frontal cortex white matter, PCA 65.7%, PC1 81.5%, PC2 22.4% (d) PCA of frontal cortex gray matter, PCA 79.9%, PC1 46.6%, PC2 26.3% (e) PCA of cerebellum white matter, PCA 58.9%, PC1 26.7%, PC2 17.6%. FW, frontal cortex white matter; FG, frontal cortex gray matter; CW, cerebellum white matter; Ctl, control

Our immunohistochemical studies suggested that there might be region-specific changes in microglial phenotype in HDLS. To explore this further and to identify HDLS-specific transcriptional changes, we performed gene expression profiling in HDLS (*n* = 6) and normal controls (*n* = 5) (Table 1). We measured expression levels of 460 macrophage-associated genes in frontal white matter (FW), frontal gray matter (FG) and cerebellar white matter (CW). To analyze transcriptomes in double-blinded fashion, we randomly assigned numbers to each individual and letters to each brain region. Hierarchical clustering based on gene expression patterns in FW was clearly different between control and HDLS, and they formed distinct clusters (Fig. 4a and b). In contrast to FW, there was no clear distinction between control and HDLS in FG or CW, suggesting that the basal microglial transcriptomic signatures were not significantly different between control and HDLS in these areas (Fig. 4a).

Principal component analysis (PCA) plots were generated to compare gene expression patterns between brain regions. Each data point on the PCA plot represents gene expression of a single region from a single case. A shorter distance between points indicates greater similarity of the transcriptome for the two cases. Not unexpectedly, the transcriptome of FG was clearly different from FW and CW (Fig. 4b). This reflects the differences in molecular profiles between microglia in white matter and gray matter. To further investigate region-specific differences in the transcriptome between HDLS and controls, PCA plots of each brain area were generated (Fig. 4c, d and e). In the figures, red symbols represent HDLS and blue symbols represent controls. Similar to heat map observations (Fig. 4a), there were clear differences between HDLS and controls in FW (Fig. 4c). In contrast, FG showed fewer differences between HDLS and controls, as well as greater sample-to-sample heterogeneity. There were also no obvious differences in transcriptome patterns in CW of HDLS and controls (Fig. 4e). Interestingly, three of the six HDLS cases had a distinctive gray matter expression pattern (Fig. 4d). These heat map and PCA map findings indicate that transcriptional changes in cerebral white matter differ from those of gray matter. Moreover, cerebellar white matter is preserved in HDLS, suggesting that white matter changes in transcriptome patterns are both disease- and region-specific.

### Enrichment analyses with differentially expressed genes in FW and FG

**Fig. 5:**
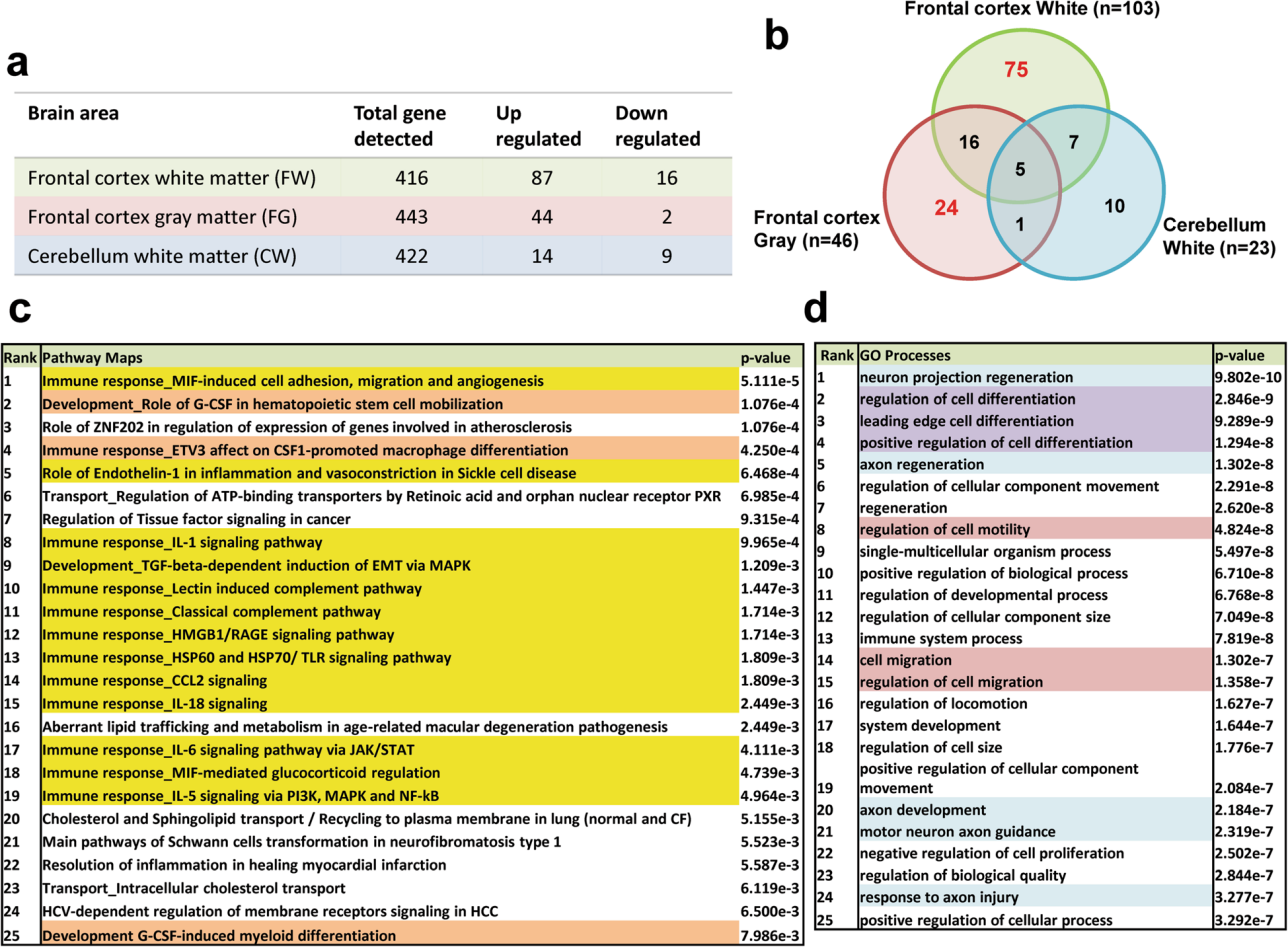
Enrichment analyses with differentially expressed genes in frontal cortex white matter. **a** The number of up- and down-regulated genes in each brain region. FW, FG and CW are frontal cortex white matter (green), frontal cortex gray matter (pink) and cerebellum white matter (blue), respectively. **b** The overlap between HDLS specific transcriptional changes in different brain areas. A Venn diagram displays the number of differentially expressed genes in HDLS. The same color code was used to indicate each brain region. The total numbers of altered genes are shown in parenthesis. Overlapping portions of circles indicates the number of genes shown in multiple brain regions. Seventy-five genes are FW-specific and 24 genes are FG-specific. **c** Enriched pathway maps in frontal cortex white matter. Enrichment analysis was performed using 75 FW-specific genes. The table with top 25 pathways displays multiple CSF-1-involved pathways and a lot of immune response-related pathways. **d** Gene ontology process in frontal cortex white matter. Enrichment analysis was performed using 75 FW-specific. The table with top 25 processes includes cell migration, cell differentiation and axonal regeneration-related processes

To further characterize region-specific molecular events in HDLS, we studied transcriptional changes in the same brain regions with greater than 1.5 fold difference and statistical significance (*p* < 0.05) (Fig. 5a). Among the three brain regions, FW showed the greatest differences in HDLS, with decreased expression of 16 and increased expression of 87 genes. In FG two genes were decreased and 44 genes were increased. In CW, nine genes were decreased and 14 genes were increased. A Venn diagram displays the overlap in differentially regulated genes by brain region (Fig. 5b). Numbers in the Venn diagram are the number of genes common to the regions that are up- or down-regulated in HDLS compared to controls.

Transcriptional changes in cerebral white matter are considered to provide information about pathogenic mechanisms at the molecular level. Thus, we performed pathway analysis using 75 genes exclusively altered in FW of HDLS to understand the molecular pathophysiology (Fig. 5c and d). We also performed pathway analysis using 24 FG genes to understand processes unaffected in HDLS (Figure S1). The names and generic functions of the 75 and 24 genes identified are summarized in Tables S1 and S2, respectively.

The enriched functions of 75 or 24 genes are listed if the *p*-value was less than 0.05 and if they had more than 2 genes in each functional category. The top 25 cerebral white matter-specific pathways and gene ontology (GO) processes are displayed in tables (Fig. 5c and d), and the top 25 cerebral gray matter-specific pathways are shown in Supplementary Data (Figure S1a and b). Interestingly, pathways involved in CSF-1 signaling are enriched only in affected FW (Fig. 5c, highlighted in orange color). In addition, many immune response-related pathways were significantly enriched in FW, but not in FG (Fig. 5c, highlighted in yellow color and Figure S1a). Several distinctive GO processes enriched in affected FW include those related to axonal function, cell differentiation and cell migration processes (Fig. 5d, highlighted in blue, purple, and pink, respectively). The GO process findings may correlate with pathologic changes observed in FW of HDLS, including the axonal damage, altered microglial phenotype and infiltration of peripheral macrophages (Figs. 1 and **2**). The GO processes related to synaptic organization, synaptic transmission and nervous system development were enriched in FG (Figure S1b, highlighted in blue color). Our results suggest that the microglial molecular signature in affected white matter clearly differs from unaffected frontal gray matter.

### Comparison of the microglial molecular signature in different brain regions

**Fig. 6:**
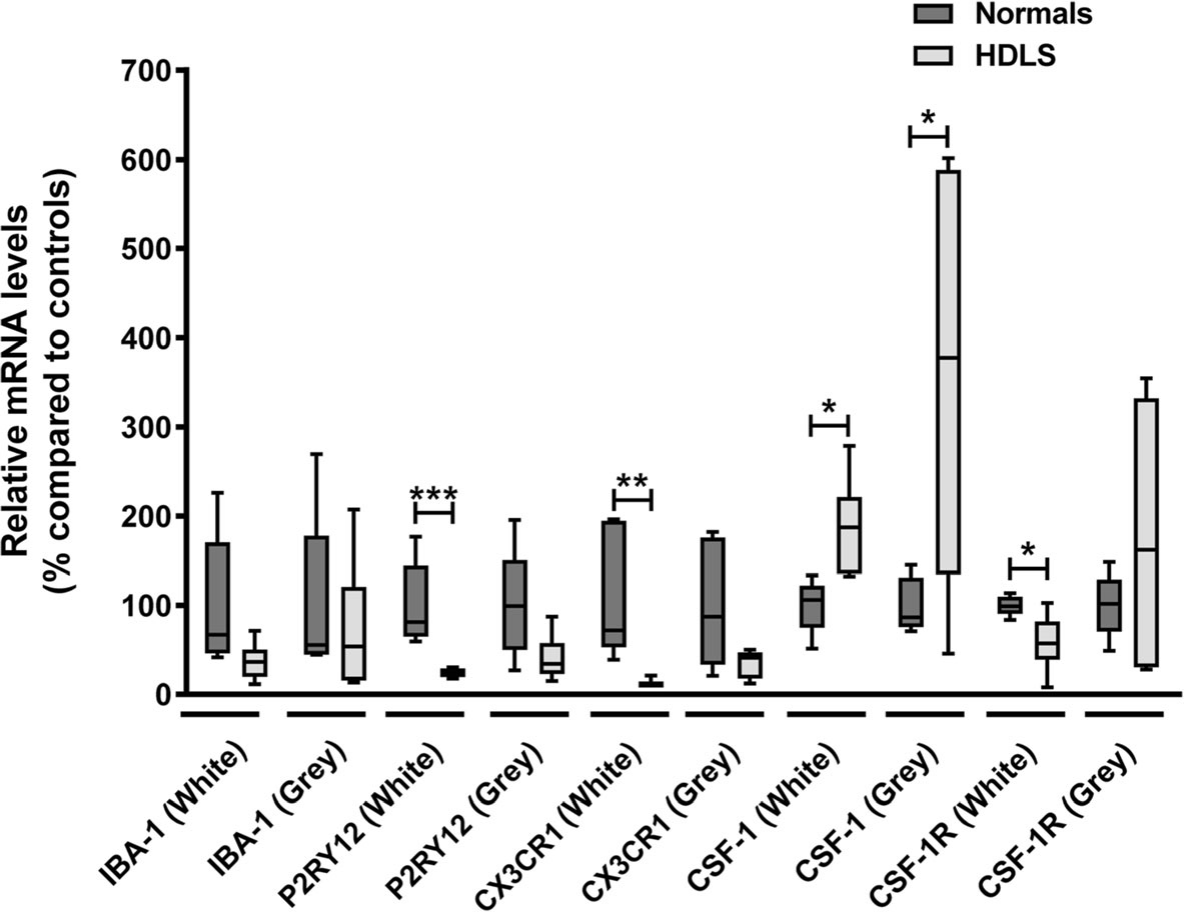
Comparison of molecular microglial signature in different brain regions of HDLS patients. The relative expression level of microglial-specific transcripts, IBA-1, P2RY12 and CX3CR1. All three genes have a trend of decreasing their level in HDLS cases. However, it is statistically significant only in frontal cortex white matter (P2RY12, *p* = 0.0033, CX3CR1, *p* = 0.0086). The relative expression level of CSF-1 and CSF-1R. The level of CSF-1 significantly increased both in white and gray matter. However, the level of CSF-1R significantly decreased only in white matter while it showed trend of increasing in gray matter. The box and whisker plot displays median values with the error bars of maximum and minimum values. Two tailed student t-test were used *p < 0.05, **p < 0.01, ***p < 0.005

We demonstrated an altered phenotype of macrophages in FW of HDLS. Abnormalities of CSF-1 signaling may explain pathologic events in cerebral white matter based on gene enrichment analysis (Fig. 5). Thus, to further characterize the molecular events in each brain region, we examined levels of microglial and CSF signaling markers in FW and FG. Expression of *IBA-1* trended to decrease in FW, but not in FG (Fig. 6). On the other hand, there were significant decreases in *P2RY12 and CX3CR1* levels in FW, yet little change in FG. Of note, *P2RY12* and *CX3CR1* are uniquely or highly expressed in microglia, whereas *IBA-1* can be detected both in microglia and monocyte-derived macrophages. Thus, decreased levels of *CX3CR1 and P2RY12* in HDLS suggest that the disease process is associated with altered phenotype of microglia in cerebral white matter microglia, but not in unaffected white matter, such as CW.

We also examined levels of *CSF-1* and *CSF-1R* in each brain region. The level of *CSF-1* was significantly increased in both FW and FG (Fig. 6). On the other hand, the level of *CSF-1R* was significantly decreased in FW, but not in FG. These results suggest that significant reduction of *CSF-1R* expression is coupled with decreased expression of microglial markers in FW. Results from gene array analysis are consistent with findings from immunohistochemistry and suggest cerebral white matter microglia have an altered phenotype in HDLS and lose their homeostatic phenotype.

## Discussion

The discovery of *CSF1R* mutations as the genetic basis of HDLS [44] was a major breakthrough in better defining this rare adult-onset hereditary disorder affecting preferentially cerebral white matter. That recessive mutations in *AARS2* [29] could produce a similar, but not identical phenotype (ALSP), suggests that more than one pathogenic process can produce cerebral white matter pathology with axonal spheroids and pigment-laden macrophages. The selectivity of certain regions of cerebral white matter in HDLS (e.g., frontal white matter) is difficult to explain given that *CSF1R* mutations should affect cells that are dependent upon CSF-1 signaling in both gray and white matter of all brain regions. Microglia clearly depend on CSF-1 signaling. A spontaneous mutant mouse (op/op [55]) with deletion of *csf1r* has bone disease due to inefficient bone remodeling by osteoclasts [55]. They also have deficient microglial functions, such as synaptic stripping in response to injury [21]. Some studies report white matter pathology and others report no overt white matter pathology in op/op mice [11, 54]. These results indicate that white matter in higher order brain regions of the human central nervous system may be uniquely dependent on microglial CSF-1 signaling. Frontal white matter is also susceptible to age-associated white matter rarefaction that appears to be closely linked to small vessel pathology [32]. Given that HDLS becomes clinically evident in adulthood, it may indicate that age-dependent processes contribute to selective vulnerability of frontal white matter when coupled with deficits in CSF1 signaling in microglia. It should be noted that although human microglia depend on CSF-1 (M-CSF) signaling, they also respond to CSF-2 (GM-CSF), especially in response to injury-associated astrocytic signaling [26]. In the absence of CSF-1R-dependent signaling, CSF-2 may compensate in normal development, but may be less efficient in response to injury with aging.

In this study, we used immunohistochemistry and mRNA expression analysis of macrophage and microglia-specific markers to assess the microglial phenotype in HDLS. We demonstrate loss of IBA-1 and P2RY12 in vulnerable white matter, but not in relatively unaffected cortical gray matter or in unaffected cerebellar white matter. Concurrent with decreased microglia-specific markers, such as P2RY12, we found increased CD68 immunoreactivity in microglia of affected white matter (Figs. 1, 2, 3). These results are in agreement with previous data in a small Japanese cohort of HDLS cases that illustrated similar pathologic hallmarks [50]. This suggests that in spite of loss of normal CSF-1 signaling due to mutations in *CSF1R*, macrophages remain abundant in HDLS, but either they have loss of the unique and homeostatic microglia phenotype or there is infiltration of peripheral macrophages. A subset of cells was positive for markers CD163 and S100, consistent with infiltration of peripheral myeloid cells and their subsequent differentiation into cells of a dendritic lineage. Dendritic cells are normally confined to the meninges, perivascular space and choroid plexus with the brain parenchyma being devoid of such cells; however, dendritic cells in the brain parenchyma are a hallmark of other inflammatory disorders such as MS. [47] The vulnerable cerebral white matter also had macrophages with an inflammatory phenotype. These changes in macrophage phenotype and the selective vulnerability of cerebral white matter were confirmed by expression array data (Figs. 4 and 5). These results highlight an aberrant and skewed inflammatory phenotype in affected frontal white matter that differed from unaffected cerebral gray matter and unaffected cerebellar white matter. A majority of white matter-specific transcriptional changes were related to immune response (Fig. 5c, highlighted in yellow). These changes in immune function were not found in unaffected cerebral gray matter. The observed region-specific changes in expression of genes related to immune functions may be downstream of haploinsufficiency of CSF-1R (Fig. 6). Based on the level of CSF-1R, the loss of homeostatic microglia in cerebral white matter may be due to significant loss of CSF-1 signaling. In contrast, cerebral gray matter was relatively spared, perhaps because CSF-1 signaling is less critical in gray matter where microglia retained their homeostatic signature (Fig. 6).

Several lines of evidence support our observation that HDLS is related to a loss of function of CSF-1R [40]. In addition, the inflammatory phenotype we observe in HDLS has been reported in other studies investigating consequences of loss of CSF-1 signaling [16, 43]. Inhibition of CSF-1R has been shown to lead to a phenotypic shift in microglia from anti-inflammatory/homeostatic to primed/activated [43]. It has been also suggested that CSF-1 and CSF-2 signaling represent opposing influences on the phenotype of macrophages, with CSF-1 being protective against inflammation [12]. Moreover, P2RY12 expression is decreased and lost in macrophages that exhibit an inflammatory phenotype [19, 31]. Taken together, the partial loss of CSF-1R signaling was associated with loss of microglial characteristics and presence of an inflammatory phenotype, which likely contributes to white matter damage in HDLS [25].

In normal brains, microglia are relatively evenly distributed throughout gray and white matter. While the cell bodies remain relatively stationary, the processes of microglia are extremely motile [34, 53]. As sentinels of the immune system, the grid-like distribution of microglia allows rapid surveillance of the brain over a wide area [34]. In our HDLS cohort, we observed an abnormal distribution of microglia, corroborating data from a previous study in a Japanese cohort [50]. In the white matter, this abnormality is in part a response to the myelin and axonal damage as microglia undergo chemotactic migration towards areas of insult. Microglia usually become heterogeneously distributed when they respond to an insult or stimulus [39]. Despite little overt neocortical pathology in HDLS, with the exception of ballooned or chromatolytic neurons [3], we also observed heterogeneous distribution of microglia in neocortex. We attempted to identify pathologic hallmarks of various insults in the regions of the gray matter that may account for this heterogeneity. The observed clusters of microglia did not appear to be associated with any obvious pathology, such as ballooned neurons, blood-brain barrier damage, tissue vacuolation, hypertrophic astrocytes or reactive astrocytosis.

It has recently been reported that CSF1R regulates microglial density and distribution in zebrafish [37], supporting our finding of altered microglial distribution in *CSF1R-*related leukoencephalopathy. Other studies have shown that the CSF-1 signaling pathway is required for microglial motility [39]. Here, we found CD68 and CD163 immunoreactive macrophages in HDLS white matter (Figs. 1 and 2). Brain parenchymal CD163 positive macrophages correlate with blood-brain barrier damage in other white matter disorders [5, 45, 56]. There is no evidence of a specific blood-brain barrier abnormality in HDLS. Parenchymal CD68- and CD163-positive cells in HDLS may be related to an altered phenotype of brain microglia or alternatively, migration of peripheral monocytes into brain parenchyma. CSF-2 promotes migration of blood-borne monocytes across the blood-brain barrier, which then differentiate into macrophages in the presence of CSF-1 and CSF-2 [16, 17, 26, 52]. CSF-2 in isolation also promotes their differentiation into a dendritic cell lineage, which maintains a phagocytic phenotype (CD68 immunopositive) [17, 30]. Thus, the reliance of HDLS patients on CSF-2 signaling due to genetically driven deficiencies in CSF-1 signaling may account for the observed cellular profiles. The co-localization of S-100 and CD68 in macrophages in HDLS suggests at least a subpopulation of the CD68 positive and IBA-1 negative cells have a dendritic cell phenotype, likely differentiating from infiltrating peripheral macrophages.

The data from our enrichment analysis support the possibility of alteration in macrophage population or distribution (Fig. 5d, highlighted in purple and pink). Enriched “regulation of cell differentiation” and “cell migration” GO processes indicate that the loss of CSF-1R may induce differentiation to alternative cell populations other than microglia or cause monocyte infiltration. Both immunohistochemistry and gene array results highlight a possible role of infiltrating monocyte-derived cells in HDLS.

Although we observed decreased gene expression of *CSF-1R* in frontal white matter, it is possible that other pathways had compensatory activation. For example, it would be informative to examine CSF-2 signaling, since it shares downstream functions in regulating differentiation of myeloid cells. The unique vulnerability of frontal white matter in HDLS may also be accounted for by differences in abundance of the two main microglial trophic factors between brain regions, with CSF-2 being significantly up-regulated in gray matter compared to white matter and vice versa for CSF-1 [26]. In addition, IL-34 is newly identified ligand for CSF-1R, and it is reported to support maintenance of microglia [14]. Recent work in a mouse *Csf1r*^*+/−*^ model illustrated a potential role in neuronal development and hypermyelination in early disease [9]. Thus, it might be interesting to further investigate whether alteration in CSF-2 or IL-34 mediated pathways account for, or compensate, the phenotypic changes in brain regions less vulnerable to pathology in HDLS.

There is evidence to suggest that a unique signature of microglia leads to a more quiescent, anti-inflammatory phenotype when microglia are cultured with CSF-1. Under these conditions, there is suppression of 19 genes, many associated with a pro-inflammatory response [7]. This homeostatic signature, termed M0, suggests that peripheral blood-borne macrophages have a more proinflammatory phenotype under baseline conditions. A neurodegenerative disease-associated microglial signature has also been described, termed MGnD [24]. MGnD represents an induced inflammatory signature mediated by the triggering receptor expressed on myeloid cells 2 (TREM2)-apolipoprotein E (APOE) pathway, which suppresses the M0 signature. Interestingly, our gene expression data from affected frontal white matter showed a significant increase in several transcripts associated with the MGnD phenotype (e.g., *APOE*, *GPNMB* and *LGALS3*) and decrease in M0 signature markers (e.g. *TMEM119*, *GPR34* and *TGFA*) and (Table S1). This result suggests that the molecular characteristics of microglia in HDLS changes with CSF-1R haploinsufficiency and that microglia in affected frontal white matter develop a MGnD phenotype. This change in brain-resident macrophage phenotype may be attributed to a change in microglial phenotype, or a loss of parenchymal microglia and a compensatory infiltration of peripheral monocytes.

Loss of the M0-homeostatic microglial signature in animal models of amyotrophic lateral sclerosis (ALS) is associated with up-regulation of inflammatory genes, which correlates with disease severity [6]. Interestingly, the phenotype of microglia we observed in HDLS is similar to that found in an SOD1 ALS mouse model and in multiple sclerosis patients, in which the molecules associated with the homeostatic microglial phenotype (e.g., P2RY12, CSF-1R) are down-regulated and inflammatory markers are up-regulated [24, 57]. This suggests that microglia are similar in these disorders and suggests other inflammatory disorders may also exhibit an MGnD phenotype when microglia lose their homeostatic signature. It has been reported that peripheral administration of anti-miR155 in SOD1 mice reversed this abnormal phenotype of microglia and ameliorated the disease [6]. Our results raise the possibility that this treatment target may have benefits in HDLS.

Recent studies have described patients with homozygous *CSF1R* mutations causing a severe leukoencephalopathy with apparent congenital absence of microglia as well as skeletal dysplasia in some patients [15, 36]. The more severe phenotype, which was associated with bone malformation, bears similarity to TREM2-associated Nasu-Hakola disease and suggests CSF-1 and TREM2 act on convergent pathways. The two disorders may lie on a disease spectrum, possibly converging via the TREM2-APOE pathway previously reported to be associated with MGnD [24]. The presence of leukoencephalopathy without apparent microglia reinforces the vital importance of microglia on white matter integrity.

In addition to mutations in *CSF1R*, some individuals with similar clinical and pathologic features have mutations in *AARS2* [29]. It is challenging to understand how a mutation in a mitochondrial transfer-RNA synthetase can lead specifically to white matter pathology. Future research into this might highlight a novel microglial phenotype regulatory system reliant on *AARS2* gene or highlight the importance of mitochondrial health in microglia and the effect this may have on their phenotype. Combined with developmental issues highlighted in *Csf1r*^*+/−*^ mice, where it has been suggested that aberrant energy metabolism may play a pathogenetic role, additional studies are warranted on these factors in HDLS [9].

Analysis of our HDLS cases revealed that age at death was younger in women than men (*p* = 0.032). The average age at death of women was 48 years, while that of men was 59 years. A sex-difference in susceptibility has been reported in a Japanese cohort of HDLS associated with *CSF1R* mutations [23]. There is a well-established sex-related link between severity and vulnerability to chronic (but not acute) inflammatory disorders in disorders such as cystic fibrosis and multiple sclerosis [8, 18]. This additional link between HDLS and autoimmune disorders implicated inflammation as a potential pathogenic mechanism and suggests that HDLS may be considered a primary microgliopathy.

Targeting CSF-1R has recently been reported to ameliorate AD pathology in APP/PS1 mice, where inhibition of CSF-1R reduced microglial proliferation and decreased the inflammatory phenotype of microglia around amyloid plaques [35]. While these short-term preclinical models might suggest that antagonism of CSF-1R may be a promising treatment in AD, given the probable need for long-term treatment and adverse response to CSF-1R inhibition may be white matter damage mediated by microglial dysfunction.

In conclusion, we defined distinct microglial populations in affected frontal white matter in HDLS with immunohistochemistry and transcriptome profiling, which differed from microglia in unaffected frontal gray matter and cerebellar white matter. A limitation of this study is that it was based on autopsy tissue where variable postmortem delay and agonal changes may have contributed to findings. On the other hand, controls were susceptible to the same shortcomings. Despite this limitation, our findings provide important insights into microglial phenotypes in affected frontal white matter providing insights into pathogenetic mechanisms of HDLS. Specifically, our study links aberrant CSF-1 signaling to region-specific loss of a homeostatic phenotype (M0) and acquisition of a phenotype (MGnD) characteristic of degenerative and demyelinating disorders. While the nature of the selective vulnerability of frontal white matter is still unknown, our study suggests that compensatory microglial signaling (e.g., CSF-2) may be deficient in frontal white matter. This may contribute to likely compensatory influx of peripheral macrophages and other myeloid-derived cells that explain both our observations of parenchymal cells of a dendritic lineage and a skewed inflammatory phenotype of the parenchymal macrophages. Our study also provides gene expression profiles of a human primary microgliopathy and suggests specific transcripts that may be protective or pathogenic. This knowledge may provide valuable information in developing therapies targeting microglial functions beyond this rare familial *CSF-1R-*related leukoencephalopathy.

## Supplementary information

**Additional file 1: Figure S1.** Enrichment analyses with differentially expressed genes in frontal cortex gray matter. (a) Enriched pathway maps in frontal cortex gray matter. Enrichment analysis was performed using 24 FG-specific genes. Unlike Fig. 6b, the table displays nothing related to CSF-1-involved pathways or immune response-related pathways. (b) Gene ontology process in frontal cortex gray matter. Enrichment analysis was performed using 24 FG-specific. The table with top 25 processes includes some synaptic function-related processes.

**Additional file 2: Figure S2.** Differentially expressed genes in frontal cortex white matter and gray matter. (a) Heat map of 75 transcripts exclusively changed in frontal cortex white matter. (b) Heat map of 24 transcripts exclusively changed in frontal cortex gray matter. The displayed groups are; FW, frontal cortex white matter; FG, frontal cortex gray matter; CW, cerebellum white matter; Ctl, control; HDLS, hereditary diffuse leukoencephalopathy with spheroids. Up- and down-regulated transcripts in HDLS are shown in red and blue, respectively.

**Additional file 3: Supplementary Table S1.** Gene names and the generic functions of 75 genes exclusively altered in frontal cortex white matter of HDLS. The gene symbol and RefSeq accession numbers are shown on the left. The genes were categorized by their generic function using MetaCore™. *P-value* was calculated by one-way ANOVA. (Blue font – up-regulated, red font – down-regulated; MGnD – transcripts positively (+) or negatively (−) associated with MGnD microglial phenotype.

**Additional file 4: Supplementary Table S2.** Gene names and the generic functions of 24 genes exclusively altered in frontal cortex gray matter of HDLS. The gene symbol and RefSeq accession numbers are shown on the left. The genes were categorized by their generic functional using MetaCore™. *p*-value was calculated by one-way ANOVA.

## Data Availability

The authors will provide upon request de-identified data, including quantitative neuropathologic data from image analyses and results of Nanostring RNA data derived from the custom MG447 human microglial chip that holds 376 microglial transcripts, 40 inflammation related transcripts, 6 positive reference genes and 8 negative controls.
