# Correction to: Loss of homeostatic microglial phenotype in CSF1R-related Leukoencephalopathy

**DOI:** 10.1186/s40478-020-00970-1

**Published:** 2020-06-24

**Authors:** Liam Kempthorne, Hyejin Yoon, Charlotte Madore, Scott Smith, Zbigniew K. Wszolek, Rosa Rademakers, Jungsu Kim, Oleg Butovsky, Dennis W. Dickson

**Affiliations:** 1 Department of Neuroscience, Mayo Clinic, 4500 San Pablo Road, Jacksonville, FL 32224 USA; 2 Institute of Neurology, University College London, London, UK; 3 Neurobiology of Disease Graduate Program, Mayo Clinic Graduate School of Biomedical Sciences, Jacksonville, FL USA; 4 Center for Neurologic Diseases, Department of Neurology, Brigham and Women’s Hospital, Harvard Medical School, Boston, MA USA; 5 Department of Neurology, Mayo Clinic College of Medicine, Jacksonville, FL USA; 6 Evergrande Center for Immunologic Diseases, Brigham and Women’s Hospital, Harvard Medical School, Boston, MA USA

**Correction to: Acta Neuropathol Commun (2020) 8:72**

**https://doi.org/10.1186/s40478-020-00947-0**

In the original publication of this article [1] the reference & citations of Chitu et al. 2020 [2] was unintentionally omitted. The original publication [1] has used data from the same tissue samples.

In this correction article the reference & citations have been published to rectify this omission.

Abstract

1. **Incorrect:***“We observed increased expression of CSF-2 in gray matter compared to affected white matter, which may contribute to selective vulnerability of white matter in HDLS”*
2. **Correct:***“Previously we observed increased expression of CSF-2 in the gray matter but not the white matter of HDLS patients****(Chitu*et al.*2020)****which may explain the selective vulnerability of white matter microglia in HDLS”.*

Discussion

**Page 11**

1. **Incorrect:***“The unique vulnerability of frontal white matter in HDLS may also be accounted for by differences in abundance of the two main microglial trophic factors between brain regions, with CSF-2 being significantly up-regulated in gray matter compared to white matter and vice versa for CSF-1 [26].”*
2. **Correct:***“The unique vulnerability of frontal white matter in HDLS may also be accounted for by differences in abundance of microglial trophic factors between brain regions. Compared to healthy individuals, ALSP patients significantly up-regulate the expression of with CSF-2 in gray matter but not in the white matter****(Chitu*et al.*2020)****and this may compensate for reduced signaling* via *the CSF-1R in microglia.”*

**Page 12**

1. **Incorrect:***“Combined with developmental issues highlighted in Csf1r+/− mice, where it has been suggested that aberrant energy metabolism may play a pathogenetic role, additional studies are warranted on these factors in HDLS [9].”*
2. **Correct:***“Combined with developmental issues highlighted in Csf1r+/− mice, where it has been suggested that aberrant energy metabolism may play a pathogenetic role, additional studies are warranted on these factors in HDLS****(Chitu*et al.*2020)****.”*
