# Supplementary figures and images for: Loss of homeostatic microglial phenotype in CSF1R-related Leukoencephalopathy
Source: Acta Neuropathol Commun. 2020 May 19;8:72. doi: 10.1186/s40478-020-00947-0 (PMC7236286; doi:10.1186/s40478-020-00947-0)

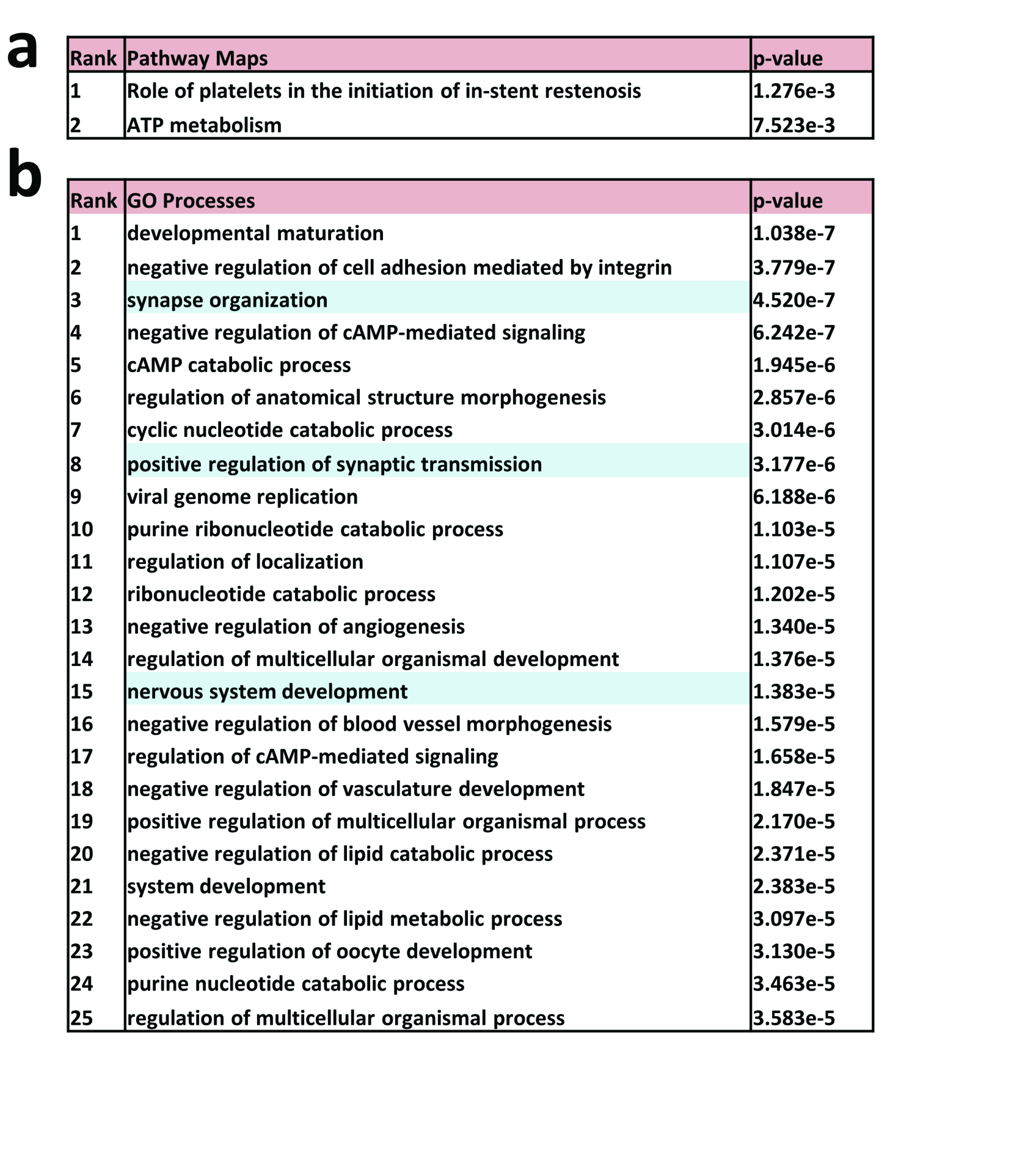

Supplement: Supplementary file 1 — Additional file 1: Figure S1. Enrichment analyses with differentially expressed genes in frontal cortex gray matter. (a) Enriched pathway maps in frontal cortex gray matter. Enrichment analysis was performed using 24 FG-specific genes. Unlike Fig. 6b, the table displays nothing related to CSF-1-involved pathways or immune response-related pathways. (b) Gene ontology process in frontal cortex gray matter. Enrichment analysis was performed using 24 FG-specific. The table with top 25 processes includes some synaptic function-related processes. [file 40478_2020_947_MOESM1_ESM.tif]

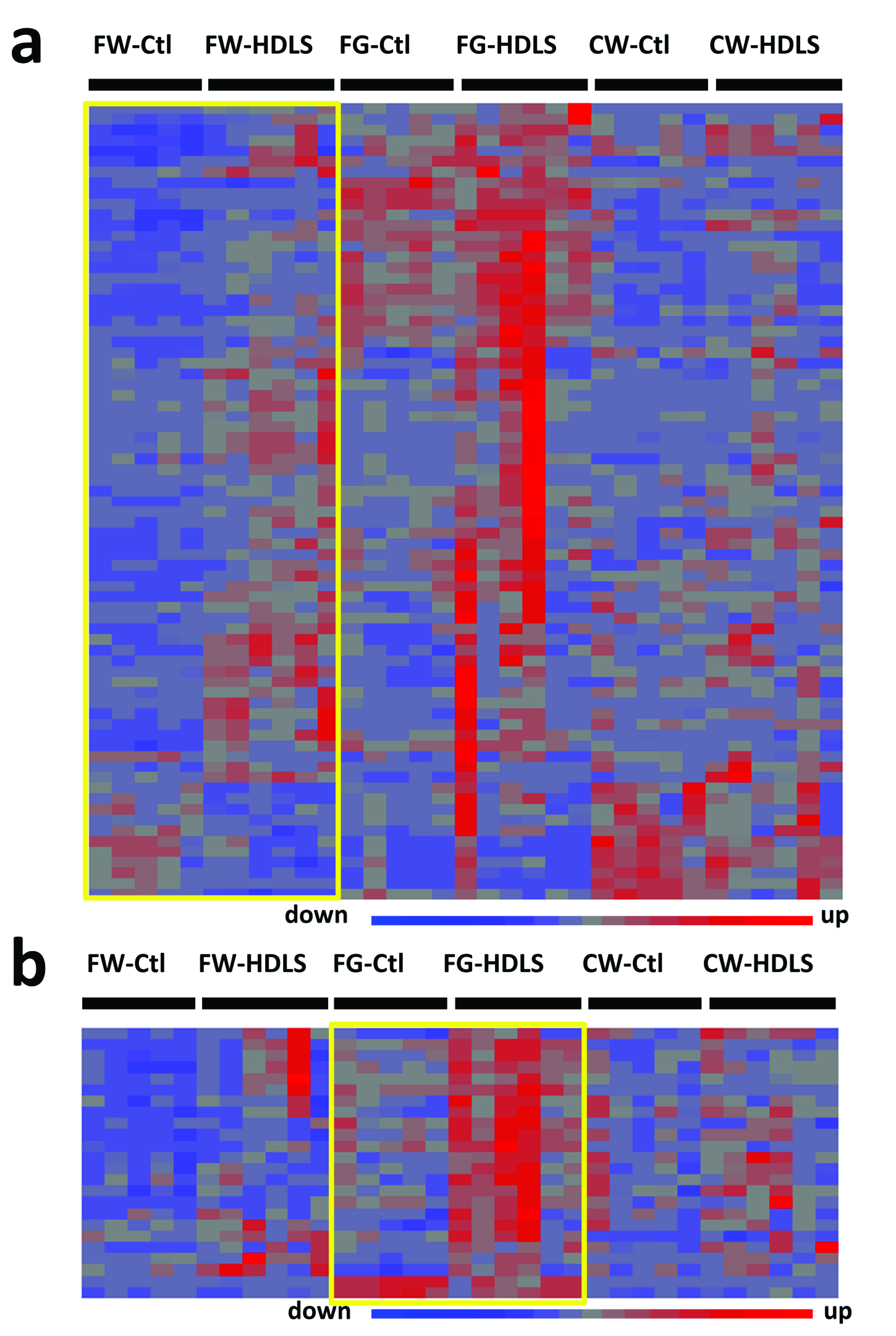

Supplement: Supplementary file 2 — Additional file 2: Figure S2. Differentially expressed genes in frontal cortex white matter and gray matter. (a) Heat map of 75 transcripts exclusively changed in frontal cortex white matter. (b) Heat map of 24 transcripts exclusively changed in frontal cortex gray matter. The displayed groups are; FW, frontal cortex white matter; FG, frontal cortex gray matter; CW, cerebellum white matter; Ctl, control; HDLS, hereditary diffuse leukoencephalopathy with spheroids. Up- and down-regulated transcripts in HDLS are shown in red and blue, respectively. [file 40478_2020_947_MOESM2_ESM.tif]
