# Supplementary material for: Loss of homeostatic microglial phenotype in CSF1R-related Leukoencephalopathy
Source: Acta Neuropathol Commun. 2020 May 19;8:72. doi: 10.1186/s40478-020-00947-0 (PMC7236286; doi:10.1186/s40478-020-00947-0)
Supplement: Supplementary file 3 — Additional file 3: Supplementary Table S1. Gene names and the generic functions of 75 genes exclusively altered in frontal cortex white matter of HDLS. The gene symbol and RefSeq accession numbers are shown on the left. The genes were categorized by their generic function using MetaCore™. P-value was calculated by one-way ANOVA. (Blue font – up-regulated, red font – down-regulated; MGnD – transcripts positively (+) or negatively (−) associated with MGnD microglial phenotype. [file 40478_2020_947_MOESM3_ESM.docx]

**Supplementary Table S1. Gene names and the generic functions of 75 genes exclusively altered in frontal cortex white matter of HDLS**

| **Gene name** | **Accession number** | **Fold Change** | **P-value** | **Functional class** | **MGnD** |
| --- | --- | --- | --- | --- | --- |
| *GPNMB* | NM_001005340.1 | 28.28 | 0.035 | Cell membrane glycoprotein | + |
| *LGALS3* | NM_001177388.1 | 6.98 | 0.010 | Binding protein | + |
| *CXCR4* | NM_003467.2 | 5.68 | 0.013 | Chemokine receptor |  |
| *SYTL1* | NM_032872.1 | 5.23 | 0.043 | Cholesterol transporter |  |
| *ABCA1* | NM_005502.2 | 5.20 | 0.003 | Ligand gated ion channel |  |
| *SPINT1* | NM_001032367.1 | 4.53 | 0.018 | Protease inhibitor |  |
| *EGR2* | NM_000399.3 | 4.23 | 0.049 | Transcription factor |  |
| *ABCC3* | NM_001144070.1 | 3.96 | 0.007 | Transporter |  |
| *APOE* | NM_000041.2 | 3.93 | 0.042 | Lipoprotein receptor | + |
| *MLPH* | NM_024101.4 | 3.73 | 0.028 | Binding protein |  |
| *SLC7A8* | NM_001267036.1 | 3.64 | 0.036 | Transporter |  |
| *HIST1H2AB* | NM_003513.2 | 3.59 | 0.027 | Histone |  |
| *CCL4* | NM_002984.2 | 3.38 | 0.042 | Chemokine ligand |  |
| *FAM46C* | NM_017709.3 | 3.24 | 0.032 | Generic protein |  |
| *C4A* | NM_007293.2 | 3.23 | 0.006 | Complement receptor |  |
| *TMEM100* | NM_018286.2 | 3.10 | 0.010 | Generic protein |  |
| *CST7* | NM_003650.3 | 3.07 | 0.039 | Binding protein |  |
| *DAB2* | NM_001343.2 | 3.07 | 0.001 | Binding protein |  |
| *PROS1* | NM_000313.1 | 2.98 | 0.005 | Receptor ligand |  |
| *CTSD* | NM_001909.3 | 2.88 | 0.024 | Cystatin F | + |
| *TMEM86A* | NM_153347.1 | 2.85 | 0.009 | Generic protein |  |
| *TRIM47* | NM_033452.2 | 2.83 | 0.006 | E3 ubiquitin ligase |  |
| *MAFB* | NM_005461.3 | 2.82 | 0.022 | Myeloid cell transcription factor | - |
| *PTMS* | NM_002824.4 | 2.80 | 0.008 | Binding protein |  |
| *SALL3* | NM_171999.2 | 2.71 | 0.002 | Transcription factor |  |
| *PTGFRN* | NM_020440.2 | 2.67 | 0.006 | Binding protein |  |
| *NAV2* | NM_001111018.1 | 2.60 | 0.001 | Binding protein |  |
| *JUN* | NM_002228.3 | 2.58 | 0.047 | Transcription factor |  |
| *HEXB* | NM_000521.3 | 2.55 | 0.004 | Generic enzyme | - |
| *SESN1* | NM_014454.2 | 2.42 | 0.028 | Generic protein |  |
| *LEPREL1* | NM_018192.2 | 2.29 | 0.007 | Generic enzyme |  |
| *GRAP* | NM_006613.3 | 2.28 | 0.032 | Binding protein |  |
| *TMEM204* | NM_024600.4 | 2.21 | 0.028 | Generic protein |  |
| *GMFB* | NM_004124.2 | 2.21 | 0.044 | Binding protein |  |
| *RNF180* | NM_001113561.1 | 2.20 | 0.033 | Binding protein |  |
| *CHST7* | NM_019886.2 | 2.11 | 0.018 | Generic enzyme |  |
| *CTNNA2* | NM_004389.2 | 2.10 | 0.046 | Binding protein |  |
| *SBF2* | NM_030962.3 | 2.08 | 0.004 | Phosphatase |  |
| *GAL3ST4* | NM_024637.4 | 2.06 | 0.027 | Generic enzyme |  |
| *LARGE* | NM_004737.4 | 2.05 | 0.007 | Generic enzyme |  |
| *USP2* | NM_171997.2 | 2.04 | 0.026 | Protease |  |
| *MAGI3* | NM_152900.2 | 1.97 | 0.035 | Kinase |  |
| *LMO2* | NM_005574.3 | 1.92 | 0.033 | Transcription factor |  |
| *ZFPM1* | NM_153813.2 | 1.89 | 0.027 | Transcription factor |  |
| *SPIRE1* | NM_001128626.1 | 1.82 | 0.002 | Binding protein |  |

**Supplementary Table S1 (continued)**

| **Gene name** | **Accession number** | **Fold Change** | **P-value** | **Functional class** | **MGnD** |
| --- | --- | --- | --- | --- | --- |
| *GBGT1* | NM_021996.4 | 1.81 | 0.032 | Generic enzyme |  |
| *FER1L3* | NM_013451.3 | 1.75 | 0.022 | Binding protein |  |
| *SEC22B* | NM_004892.5 | 1.72 | 0.042 | Binding protein |  |
| *RAB3IL1* | NM_013401.2 | 1.70 | 0.045 | Regulators |  |
| *EXTL3* | NM_001440.3 | 1.70 | 0.046 | Generic enzyme |  |
| *CMKLR1* | NM_004072.1 | 1.67 | 0.020 | GPCR |  |
| *KCNMA1* | NM_001014797.2 | 1.65 | 0.031 | Voltage gated ion channel |  |
| *ST3GAL6* | NM_006100.2 | 1.65 | 0.010 | Generic enzyme |  |
| *NEDD1* | NM_001135175.1 | 1.60 | 0.022 | Generic protein |  |
| *ABI1* | NM_001012750.1 | 1.53 | 0.022 | Binding protein |  |
| *SLC6A1* | NM_003042.3 | -1.55 | 0.047 | Transporter |  |
| *TMEM144* | NM_018342.4 | -1.75 | 0.004 | Generic protein |  |
| *APBB2* | NM_173075.4 | -1.79 | 0.037 | Binding protein |  |
| *PALD1* | NM_014431.2 | -1.87 | 0.015 | Phosphatase |  |
| *TGFA* | NM_003236.2 | -1.92 | 0.009 | Receptor ligand | - |
| *GPR34* | NM_005300.2 | -1.95 | 0.029 | GPCR | - |
| *TMEM119* | NM_181724.2 | -2.05 | 0.024 | Generic protein |  |
| *ENPP2* | NM_001040092.2 | -2.23 | 0.022 | Generic enzyme |  |
| *TMEM206* | NM_018252.2 | -2.34 | 0.019 | Generic protein |  |
| *OMG* | NM_002544.3 | -2.60 | 0.007 | Binding protein |  |
| *SLC2A5* | NM_003039.2 | -2.98 | 0.018 | Transporter |  |
