# Supplementary material for: Loss of homeostatic microglial phenotype in CSF1R-related Leukoencephalopathy
Source: Acta Neuropathol Commun. 2020 May 19;8:72. doi: 10.1186/s40478-020-00947-0 (PMC7236286; doi:10.1186/s40478-020-00947-0)
Supplement: Supplementary file 4 — Additional file 4: Supplementary Table S2. Gene names and the generic functions of 24 genes exclusively altered in frontal cortex gray matter of HDLS. The gene symbol and RefSeq accession numbers are shown on the left. The genes were categorized by their generic functional using MetaCore™. p-value was calculated by one-way ANOVA. [file 40478_2020_947_MOESM4_ESM.docx]

**Supplementary Table S2. Gene names and the generic functions of 24 genes exclusively altered in frontal cortex gray matter of HDLS**

| **Gene name** | **Accession number** | **Fold Change** | **P-value** | **Generic functional class** |
| --- | --- | --- | --- | --- |
| *SPSB1* | NM_025106.3 | 2.97 | 0.015 | Receptor |
| *CCL2* | NM_002982.3 | 2.94 | 0.047 | Receptor ligand |
| *HVCN1* | NM_001040107.1 | 2.93 | 0.034 | Voltage gated ion channel |
| *CARHSP1* | NM_001042476.1 | 2.78 | 0.029 | Binding protein |
| *PDGFA* | NM_002607.5 | 2.22 | 0.046 | Receptor ligand |
| *DNAJB4* | NM_007034.3 | 2.05 | 0.038 | Binding protein |
| *NFIA* | NM_005595.1 | 1.97 | 0.026 | Transcription factor |
| *TGM1* | NM_000359.2 | 1.92 | 0.049 | Generic enzyme |
| *CADM1* | NM_014333.3 | 1.79 | 0.010 | Binding protein |
| *JARID2* | NM_004973.2 | 1.78 | 0.037 | Transcription factor |
| *TMEM47* | NM_031442.3 | 1.75 | 0.011 | Generic protein |
| *PDE3B* | NM_000922.3 | 1.75 | 0.011 | Generic enzyme |
| *CTTNBP2NL* | NM_018704.2 | 1.72 | 0.029 | Generic protein |
| *PTPRZ1* | NM_002851.2 | 1.72 | 0.048 | Generic protein |
| *AHCYL1* | NM_006621.4 | 1.72 | 0.021 | Generic enzyme |
| *PLA2G15* | NM_012320.3 | 1.70 | 0.028 | Phospholipase |
| *SLC1A3* | NM_004172.4 | 1.62 | 0.032 | Transporter |
| *JMY* | NM_152405.2 | 1.58 | 0.031 | Binding protein |
| *MECP2* | NM_001110792.1 | 1.57 | 0.036 | Binding protein |
| *ARHGAP5* | NM_000476.2 | 1.53 | 0.016 | Regulators |
| *AK1* | NM_000476.2 | 1.52 | 0.046 | Kinase |
| *GOLM1* | NM_016548.3 | 1.52 | 0.037 | Generic protein |
| *UNC13A* | NM_001080421.2 | -1.58 | 0.042 | Generic protein |
| *NEFL* | NM_006158.3 | -1.77 | 0.033 | Binding protein |
